# The association of myocardial strain with cardiac magnetic resonance and clinical outcomes in patients with acute myocarditis

**DOI:** 10.3389/fcvm.2023.1121083

**Published:** 2023-07-31

**Authors:** Alexandre M. Soeiro, Aline S. Bossa, Maria C. César, Tatiana C. A. T. Leal, Guilherme Garcia, Rafael A. Fonseca, Débora Nakamura, Patrícia O. Guimarães, Maria C. F. A. Soeiro, Carlos V. Serrano, Paulo R. Soares, Christian Mueller, Alexandre Mebazaa, Fábio Fernandes, Cesar H. Nomura, Carlos E. Rochitte, Múcio T. de Oliveira

**Affiliations:** ^1^ Heart Institute, InCor, University of Sao Paulo Medical School, Sao Paulo, Brazil; ^2^ Cardiovascular Research Institute Basel (CRIB) and Department of Cardiology, University Hospital Basel, University of Basel, Basel, Switzerland; ^3^ University Paris Diderot, Paris, France; ^4^ Department of Anaesthesia and Critical Care, University Hospitals Saint Louis-Lariboisière, Paris, France

**Keywords:** magnetic resoance imaging, pericarditis, myocarditis, myocardial strain, diagnosis

## Abstract

**Introduction:**

The role of myocardial strain in risk prediction for acute myocarditis (AMC) patients, measured by cardiac magnetic resonance (CMR), deserves further investigation. Our objective was to evaluate the association between myocardial strain measured by CMR and clinical events in AMC patients.

**Material and methods:**

This was a prospective single-center study of patients with AMC. We included 100 patients with AMC with CMR confirmation. The primary outcome was the composite of all-cause mortality, heart failure and AMC recurrence in 24 months. A subgroup analysis was performed on a sample of 36 patients who underwent a second CMR between 6 and 18 months. The association between strain measures and clinical events or an increase in left ventricular ejection fraction (LVEF) was explored using Cox regression analysis. Global peak radial, circumferential and longitudinal strain in the left and right ventricles was assessed. ROC curve analysis was performed to identify cutoff points for clinical event prediction.

**Results:**

The mean follow-up was 18.7 ± 2.3 months, and the composite primary outcome occurred in 26 patients. The median LVEF at CMR at baseline was 57.5% (14.6%). LV radial strain (HR = 0.918, 95% CI: 0.858–0.982, *p* = 0.012), LV circumferential strain (HR = 1.177, 95% CI: 1.046–1.325, *p* = 0.007) and LV longitudinal strain (HR = 1.173, 95% CI: 1.031–1.334, *p* = 0.015) were independently associated with clinical event occurrence. The areas under the ROC curve for clinical event prediction were 0.80, 0.79 and 0.80 for LV radial, circumferential, and longitudinal strain, respectively. LV longitudinal strain was independently correlated with prognosis (HR = 1.282, CI 95%: 1.022–1.524, *p* = 0.007), even when analyzed together with ejection fraction and delayed enhancement. LV and right ventricle (RV) strain were not associated with an increase in LVEF. Finally, when the initial CMR findings were compared with the follow-up CMR findings, improvements in the measures of LV and RV myocardial strain were observed.

**Conclusion:**

Measurement of myocardial strain by CMR can provide prognostic information on AMC patients. LV radial, circumferential and longitudinal strain were associated with long-term clinical events in these patients.

## Introduction

Most patients with acute myocarditis (AMC) should be treated conservatively. The use of standard heart failure (HF) therapies such as diuretics, vasodilators, beta-blockers and aldosterone antagonists is indicated only in cases with reduced ejection fraction. When cardiogenic shock is present, the use of inotropes, intravenous vasodilators and circulatory assist devices may be needed, and progressive improvement is usually seen (1–5). The initial clinical presentation with low cardiac output syndrome, left ventricle ejection fraction <50%, the presence of inflammation during immunohistological analysis and the absence of treatment with beta-blockers are the factors most closely correlated with death and the need for heart transplantation within 5 years (3, 4, 6, 7).

The use of cardiac magnetic resonance (CMR) has increased the confirmation rates of AMC by detecting hypersignals in T2 (inflammation marker) and late gadolinium enhancement imaging (1). The diagnostic criteria for AMC by CMR were defined by the Lake Louise Criteria: AMC can be confirmed if at least one T2-based criterion (global or regional increase in myocardial T2 relaxation time or an increased signal intensity in T2-weighted CMR images) is found along with at least one T1-based criterion (increased myocardial T1, extracellular volume, or late gadolinium enhancement) (5, 8, 9).

In recent years, efforts have been made to identify CMR findings correlated with prognosis in patients with AMC (10–15). However, data on whether quantitative strain measurements by CMR may have a role in risk prediction for future adverse outcomes in AMC patients are recent. The present study aims to evaluate the association between myocardial strain measures obtained by CMR and the occurrence of clinical events in patients with AMC. Additionally, the association between myocardial strain and changes in the left ventricular ejection fraction (LVEF) was assessed in a subgroup of patients who underwent a second CMR test during the follow-up.

## Material and methods

### Study patients

**Figure 1:**
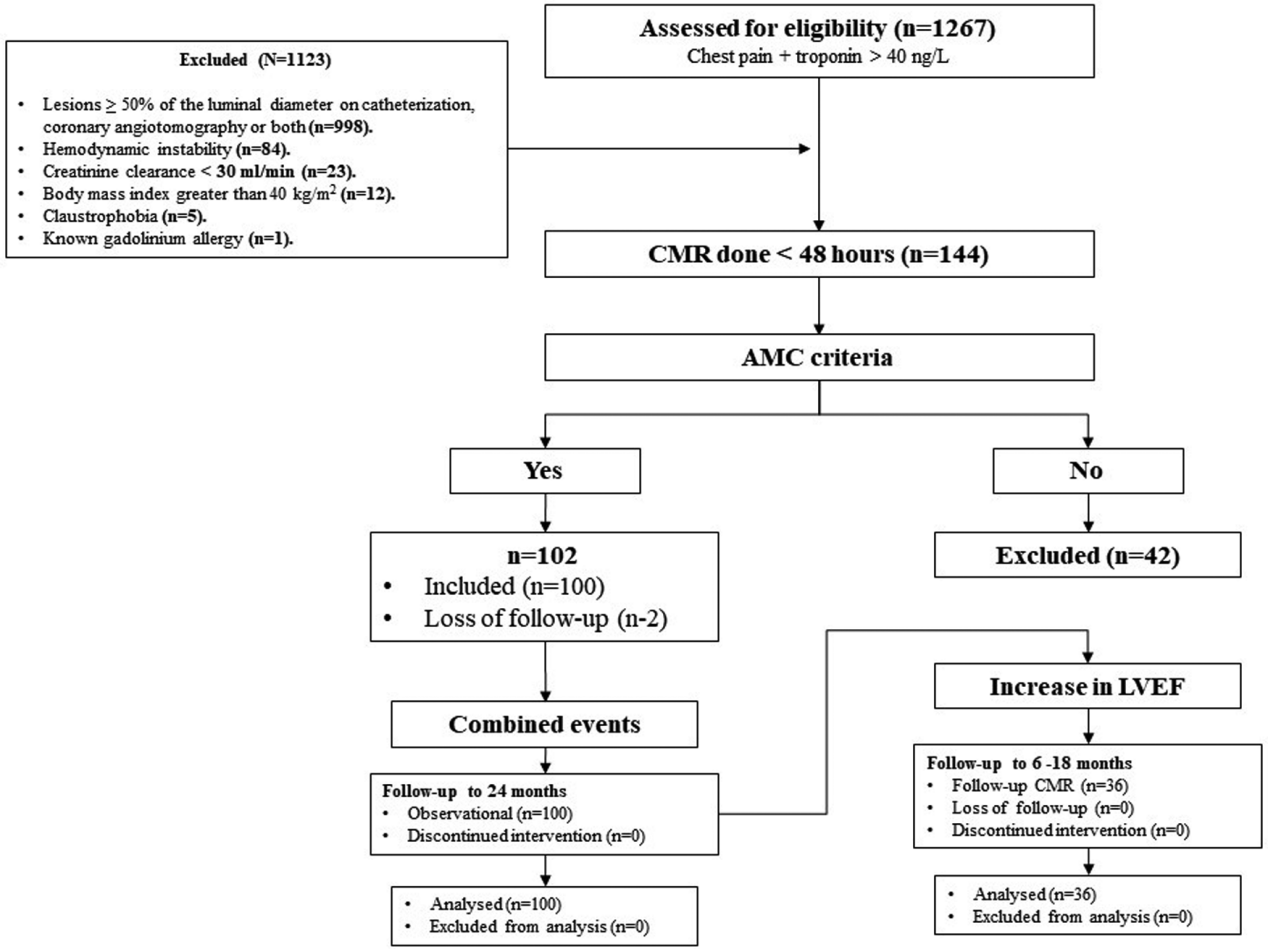
Study design and flowchart. CMR, cardiac magnetic resonance; AMC, acute myocarditis.

The study design is presented in Figure 1. This was a prospective single-center study of patients with AMC. Patients were recruited from the Emergency Department of the Heart Institute, InCor, University of Sao Paulo Medical School, Sao Paulo, Brazil. To be included, patients had to be 18 years or older, present with chest pain and/or electrocardiographic changes associated with troponin elevation (above the 99th percentile) in the absence of coronary artery stenosis (lesions <50% of the luminal diameter on invasive or coronary computed tomography angiography) and meet the diagnostic criteria for AMC, which were the presence of myocardial late gadolinium enhancement and/or myocardial edema on CMR <48 h after admission (2, 7, 9). The exclusion criteria were pregnancy, hemodynamic instability (pulmonary congestion/systolic blood pressure less than 90 mmHg), body mass index greater than 40 kg/m^2^, creatinine clearance <30 ml/min (Cockcroft and Gault), a known gadolinium allergy and claustrophobia. Our institutional review board for human subject studies approved this study, and all participants provided written informed consent prior to enrollment. A subgroup analysis was performed with a sample of patients who underwent a second CMR between 6 and 18 months after the initial episode of AMC. High-sensitivity cardiac troponin I was measured by the commercial ADVIA Centaur® TnI-Ultra kit (Siemens Healthcare Diagnostics, Tarrytown, NY, USA). The 99th-percentile value was 40 ng/L.

### Cardiac magnetic resonance

All patients underwent a brief interview prior to the CMR test, which covered height, weight, medical history and previous exams. CMR exams were performed on all patients with a scanner of 1.5 T (*Vantage/Titan, Toshiba Medical Systems, Otawara, Japan).* Images were acquired and coupled to the ECG during breath-hold in the four-chamber, short-axis and long-axis views of the left ventricle (LV) in the exact same location in different sequences. This allowed a precise comparison between cardiac function and regional myocardial structure. The parameters used in the dynamic sequences were a repetition time (RT) of 3.5 ms, echo time (ET) of 1.5 ms, flip angle of 60°, receiver bandwidth of ±125 kHz, field of view (FOV) of 35 cm × 35 cm, matrix of 256 × 148, temporal resolution of 35 ms, and slice thickness of 8.0 mm, without a gap between the slices.

For the detection of edema, T2-weighted images were acquired with a triple inversion-recovery pulse fast spin‒echo (FSE) (dark blood) and long echo time (ET > 70 ms, 100 ms ideally), with breath-hold, in short-axis view of the LV. The images were positioned carefully to guarantee the presence of adequate skeletal muscle for reference. In this sequence, we used volumetric body coil acquisition to guarantee volume homogeneity and avoid signal loss, as usually occurs with surface coils. The parameters evaluated were an RT of 2× the RR interval, an ET of 80–120 ms, an echo train length of 24 (ETL), an inversion time (IT) of 140 ms, a slice thickness of 10 mm, a gap of 2 mm, a field of view of 34 cm × 38 cm, and a matrix of 256 × 256. We applied a spin‒echo free breathing sequence in the axial plane tipped inferiorly toward the left to align with the inferior wall of the left ventricle, with no change in acquisition parameters from before to immediately after intravenous injection of 0.2 mmol/kg gadolinium contrast agent (Gadovist®, Bayer Schering Pharma©, Berlin, Germany) at a 3 ml/s rate, which was followed by 30 ml of saline. The sequence began immediately after injection and lasted 3–4 min; the images reflected gadolinium enhancement for an average time of 2 min. The sequence parameters were as follows: field of view adjusted to include the left upper arm, echo train length of 2–4, slice thickness of 8–10 mm, 128 *y*-lines, and four signal averages.

The myocardial late enhancement technique was used to investigate myocardial fibrosis. An inversion-recovery prepared gradient-echo was acquired 10–20 min after the contrast was used, with the following parameters: RT, 7.1 ms; ET, 3.1 ms; flip angle, 20°; cardiac phases, 1; views per segment, 16–32; matrix, 256 × 192; slice thickness, 8 mm; gap between slices, 2 mm; field of view, 32–38 cm; inversion time, 150–250 ms; receiver bandwidth, 31.25 kHz; number of excitations, 2; and acquisition every heart beat. Additionally, we performed a sequence as described above but with a TI of 600 ms for further characterization of cardiac thrombus.

The late gadolinium enhancement area was calculated using a threshold technique with a threshold value of 2 standard deviations using cvi software. That is, all myocardial pixels with an enhancement 2 standard deviations above the mean of the normal myocardium were considered to have late enhancement. We delimited the endocardium and epicardium manually so that the software did the calculations only in the myocardium and excluded any evident visual artifacts or pixels that may be external to the myocardium (epicardial fat or cavity).

**Figure 2:**
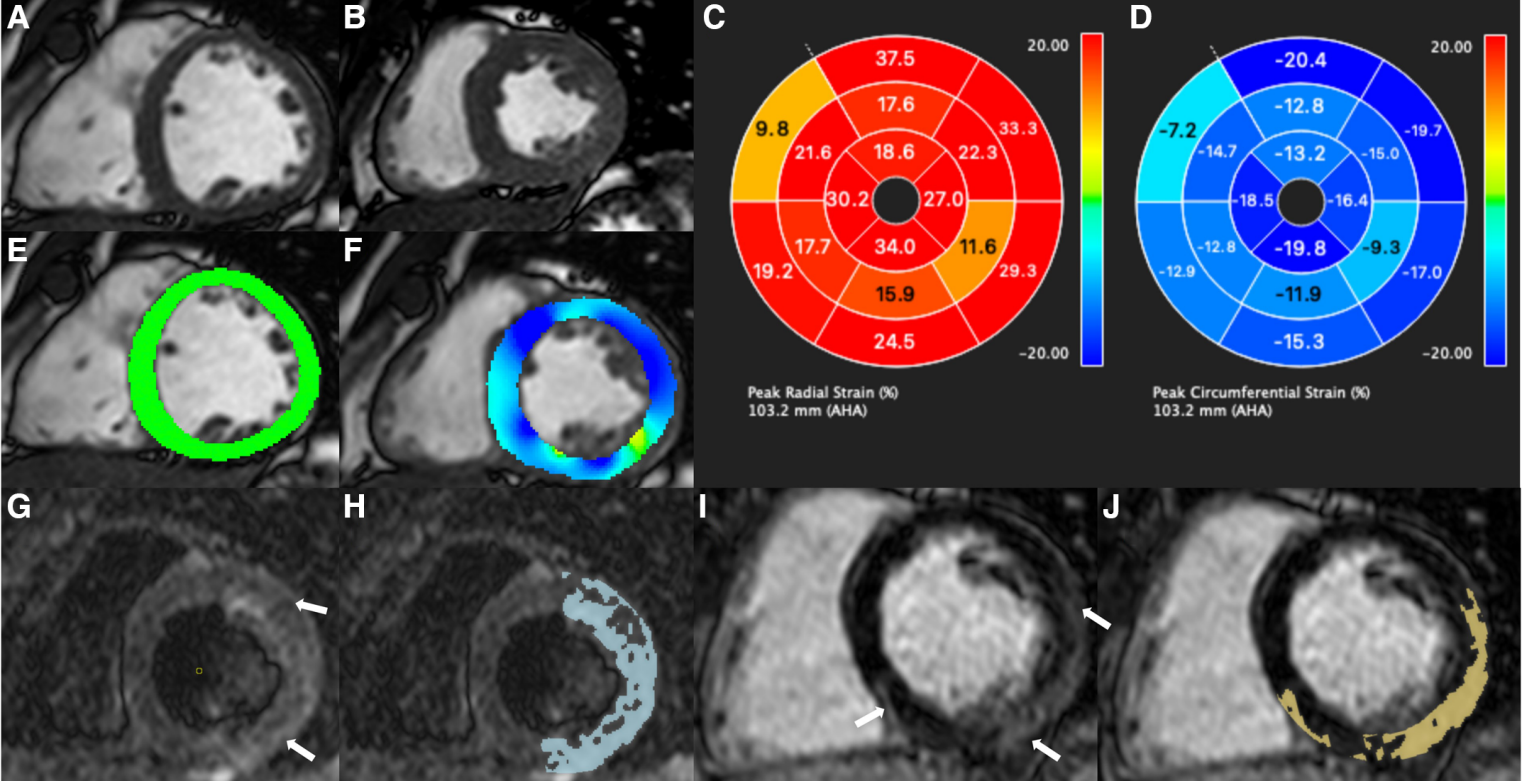
In (**A,B**) cine short axis in medial cut in diastole (**A**) and systole (**B**). (**C,D**) Bullseye radial strain and circumferential strain, respectively. The endocardial and epicardial were delimited manually in diastole in short-axis cine images in medial location of the left ventricle with automatic propagation to the entire cardiac cycle. Circumferential strain in the short axis cine in medial location of the LV in diastole (**E**) and systole (**F**) with contractile defect in the inferior, inferolateral and attenuated contraction in the septal and anterior region, corroborating with the bullseye (**C,D**). In (**G**) T2-weighted image in medial section of the LV showing edema (white arrows) and quantification (blue areas). Image (**I**) showed delayed short-axis enhancement with myocardial fibrosis (white arrows) and quantification (yellow areas).

Myocardial strain analyses were performed using cvi 42 software version 5.13.5 (*Circle Cardiovascular Imaging, Inc., Calgary, Canada*). The global radial, circumferential and longitudinal peak strains of the LV and right ventricle (RV) were acquired through the short-axis view across 4 chambers and 3 chambers. In the tissue tracking analysis, the endocardial and epicardial borders were manually contoured at the end of diastole. The parameters were automatically calculated by the software. In the subgroup of patients undergoing a second CMR, the same methodology described above was used. In all measurements, global strain was used. For the longitudinal strain, the long axis (LAX slices) was used. For the radial and circumferential strain, the short axis (SAX slices) was used. The same measurements were performed in the right and left ventricles. In the right ventricle, measurements were performed with greater caution due to the lower thickness of the ventricle wall. Examples of the strain measures are shown in Figure 2.

All analyses were performed blinded to patient baseline characteristics and outcomes. CMR strain measurements were performed independently in a second step by 2 level 3 accredited CMR clinicians, where readers were further blinded to all other CMR findings, including late enhancement, edema and LVEF and outcomes.

### Study outcomes

The primary outcome of this study was the composite of death from all causes, HF and AMC recurrence. HF was defined as hospitalization associated with the disease or symptoms with functional class ≥2 according to the New York Heart Association classification (3). Recurrence of AMC was present when the patient presented with a new event that fulfilled the diagnostic criteria for AMC established in the guidelines (3, 4).

Within the subgroup of patients who underwent a second CMR during follow-up, an increase in LVEF was defined as an increase ≥5% in LVEF in the second CMR over the first CMR.

### Clinical follow-up

Hospitalization data from all patients were obtained according to medical records after admission. After hospital discharge, the patients were followed via medical visits that occurred every 6 months, according to the standard local practice at the study site for up to 24 months. Patients who did not return for these visits for any reason during this time period received phone calls to assess survival status and the occurrence of clinical events.

### Statistical analysis

Descriptive analysis of baseline characteristics are reported as mean and standard deviation for variables with a normal distribution and median and interquartile interval for those with a nonnormal distribution. The comparison between patients who presented with clinical events and those who did not was performed using *Q*-square for categorical variables. For continuous variables, when the Kolmogorov‒Smirnov normality test showed a normal distribution, the unpaired *t*-test was used. If the distribution was not normal, the Mann‒Whitney *U*-test was used. A *p*-value less than 0.05 was considered statistically significant.

The associations between CMR peak strain in the radial, circumferential and longitudinal axes in the LV and RV and the occurrence of clinical events were explored. Additionally, the association between CMR peak strain and changes in LVEF was described. Multivariate analysis was performed by Cox regression analysis only when there was a significant association (*p* < 0.05) between the variable and the occurrence of clinical events in the univariate analysis. The following baseline characteristics were considered as variables in the analysis because they were significantly different between the groups: age, sex, diabetes mellitus, systemic arterial hypertension, dyslipidemia, smoking, creatinine and medications used at hospital discharge.

Analysis by receiver operating characteristic (ROC) curves was also performed to identify the cutoff points with the highest discrimination for clinical event prediction. The 95% confidence intervals (CIs) of the cutoff points are shown. For direct comparisons of continuous variables in the initial vs. follow-up CMR, the Wilcoxon test was used. Data were analyzed with SAS Statview 5.0 software.

## Results

### Study population

A total of 102 patients presenting with chest pain and a final diagnosis of AMC confirmed by CMR were included between March 2013 and June 2018 at the Emergency Department of the Heart Institute, InCor, University of Sao Paulo Medical School, Sao Paulo, Brazil. Two patients were lost to follow-up during the study period. The mean follow-up was 18.7 ± 2.3 months. The final analysis included the remaining 100 patients. The baseline characteristics are presented for the overall population and stratified by the occurrence of clinical events in Table 1. The mean age was 37.9 ± 18.1 years, and 82% of participants were male. The most prevalent cardiovascular risk factor was systemic arterial hypertension, in 20% of patients. The median high-sensitivity cardiac troponin level was 10,300.0 (2,200.0) ng/L, and the median LVEF by CMR at baseline was 57.5% (14.6%). The average time between the onset of symptoms and patient arrival was 21.3 ± 1.8 h. The average time from the arrival of the patient to the performance of the CMR and the beginning of the specific treatment were 14.1 ± 1.4 h and 17.8 ± 2.1 h, respectively. The main findings described in the initial CMR are shown in Table 2. The myocardial strain values found in the overall patients were: LV radial strain = 23.2 ± 8.2, LV circumferential strain = −14.5 (−17.3), LV longitudinal strain = −13.4 ± −3.8, RV radial strain = 28.4 ± 13.3, RV circumferential strain = −17.1 ± 7.5, RV longitudinal strain = −21.5 (23.0).

**Table 1:** Clinical characteristics of patients stratified by the occurrence of clinical events.

Baseline characteristics	Overall (*n* = 100)	Composite events		*p*
		Yes (*n* = 26)	No (*n* = 74)	
Age	37.9 ± 18.1	45.7 ± 22.8	34.9 ± 15.5	0.009
Male sex (%)	82 (80.4%)	17 (65.4%)	64 (86.5%)	0.018
Diabetes mellitus (%)	2 (2.0%)	2 (7.7%)	0	0.016
Hypertension (%)	20 (19.6%)	8 (30.7%)	12 (16.2%)	0.111
Smoking (%)	16 (15.7%)	3 (11.5%)	12 (29.7%)	0.566
Dyslipidemia (%)	12 (11.8%)	5 (19.2%)	6 (8.1%)	0.119
Troponin (ng/L)	10,300 (2,200)	13,900 (18,500)	19,200 (18,300)	0.012
Creatinine (mg/dl)	1.0 ± 0.3	1.2 ± 0.5	0.9 ± 0.2	0.002
LVEF (%)	57.5 (14.6)	40.4 (18.8)	57.7 (9.6)	<0.0001
CRP (mg/dl)	22.4 (6.7)	33.0 (47.5)	48.3 (63.1)	0.085
BNP (pg/ml)	93.5 (22.5)	849.0 (795.6)	118.6 (202.9)	0.001
Medications used at hospital discharge				
Beta-blocker (%)	43 (42.2%)	19 (73.1%)	24 (32.4%)	<0.0001
Spironolactone (%)	20 (19.6%)	10 (38.5%)	10 (13.5%)	0.006
NSAID (%)	41 (40.2%)	3 (11.5%)	36 (48.6%)	0.001
Colchicine (%)	28 (27.5%)	2 (7.7%)	26 (35.1%)	0.007
ACEI/ARB (%)	48 (47.1%)	19 (73.1%)	29 (39.2%)	0.003

Variables described as mean + standard deviation or median (interquartile range); LVEF, left ventricle ejection fraction by CMR; CRP, C-reactive protein; BNP, B-type natriuretic peptide; NSAID, nonsteroidal anti-inflammatory; ACEI, angiotensin converting enzyme inhibitors; ARB, angiotensin receptor blockers.

**Table 2:** Main findings described in the initial CMR in 100 patients with acute myocarditis.

LVEF (%)	57.5 (14.6)
RVEF (%)	53.0 (11.9)
Left ventricle end systolic volume (ml)	61.5 (44.0)
Left ventricle end diastolic volume (ml)	140.0 (67.5)
Right ventricle end systolic volume (ml)	57.5 (34)
Right ventricle end diastolic volume (ml)	117.6 (91.8)
Positive T2 hypersignal (%)	81 (81%)
Pericardial effusion (%)	34 (33.3%)
Presence of thrombus (%)	2 (2.0%)
LGE area (%)	18.2 + 13.4
Positive LGE (%)	96 (96%)
Pattern of LGE	
Mid-wall	94 (94%)
Subepicardial	20 (20%)
Transmural	0
Subendocardial	0
Location of LGE	
Apical anterior	17 (17%)
Mild anterior	24 (24%)
Basal anterior	16 (16%)
Mild anteroseptal	20 (20%)
Basal anteroseptal	24 (24%)
Apical septal	18 (18%)
Mild inferoseptal	19 (19%)
Basal inferoseptal	17 (17%)
Apical inferior	15 (15%)
Mild inferior	23 (23%)
Basal inferior	20 (20%)
Mild inferolateral	38 (38%)
Basal inferolateral	37 (37%)
Apical lateral	32 (32%)
Mild anterolateral	32 (32%)
Basal anterolateral	29 (29%)
Apex	14 (14%)

Variables described as mean ± standard deviation or median (interquartile range); LVEF, left ventricle ejection fraction; RVEF, right ventricle ejection fraction; LGE, late gadolinium enhancement.

### Clinical outcomes

There were 2 deaths during follow-up. HF events were observed in 20 patients, and AMC recurrence was observed in 17 patients. The composite primary outcome occurred in 26 patients.

### Associations between myocardial strain in the initial CMR and clinical events

The results of the univariate analysis are presented in Table 3. LV radial strain, LV circumferential strain, LV longitudinal strain, and RV longitudinal strain were associated with the occurrence of clinical events. In the adjusted model, LV radial strain (HR = 0.918, 95% CI: 0.858–0.982, *p* = 0.012), LV circumferential strain (HR = 1.177, 95% CI: 1.046–1.325, *p* = 0.007) and LV longitudinal strain (HR = 1.173, 95% CI: 1.031–1.334, *p* = 0.015) remained associated with clinical events.

**Table 3:** Univariate and multivariate associations between myocardial strain measures and clinical events.

	Composite clinical events							
	Univariate analysis					Mutivariate analysis		
	Yes	No	HR	CI 95%	*p*	HR	CI 95%	*p*
LV radial strain (%)	16.9 ± 8.6	25.0 ± 6.9	0.843	0.773–0.919	<0.0001	0.918	0.858–0.982	0.012
LV circumferential strain (%)	−11.1 (4.5)	−15.4 (2.8)	1.434	1.209–1.701	<0.0001	1.177	1.046–1.325	0.007
LV longitudinal strain (%)	−10.2 ± 4.3	−14.4 ± 2.9	1.420	1.207–1.670	<0.0001	1.173	1.031–1.334	0.015
RV radial strain (%)	24.8 ± 10.9	29.1 ± 13.5	0.974	0.939–1.010	0.154			
RV circumferential strain (%)	−9.5 ± 6.0	−10.9 ± 4.7	1.054	0.936–1.154	0.249			
RV longitudinal strain (%)	−15.1 (4.9)	−17.8 (8.2)	1.044	1.001–1.106	0.007	0.987	0.936–1.042	0.642

Variables described as mean ± standard deviation or median (interquartile range); LV, left ventricle; RV, right ventricle; HR, hazard ratio; CI, confidence interval.

**Figure 3:**
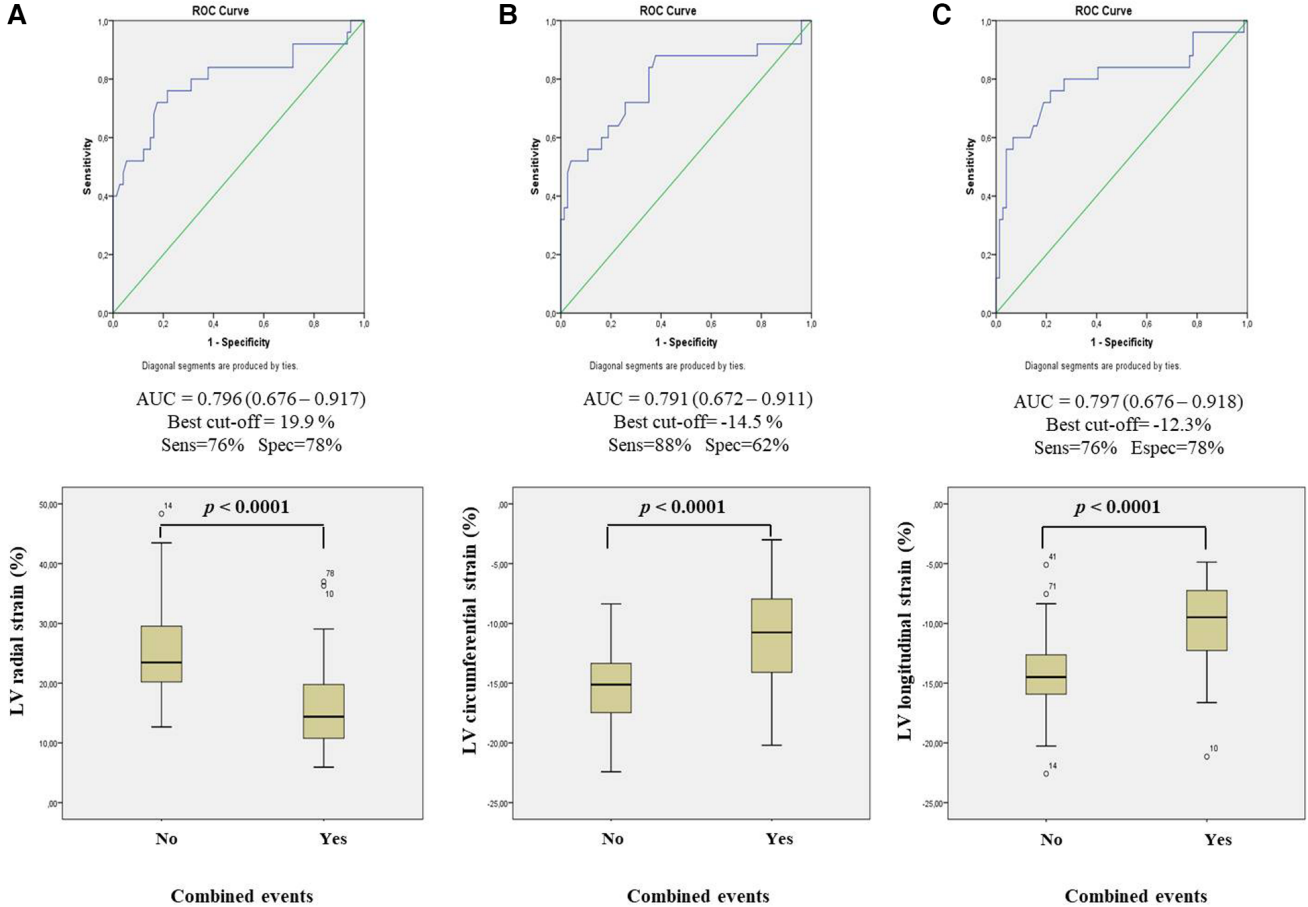
ROC curve and boxplot diagrams relating combined events with (**A**) LV radial strain; (**B**) LV circumferential strain; and (**C**) LV longitudinal strain related. LV, left ventricle; AUC, area under curve; sens, sensitivity; spec, specificity.

The cutoff points with the highest discrimination for clinical event prediction were 19.9% for LV radial strain, −14.5% for LV circumferential strain, and −12.3% for LV longitudinal strain. The areas under the ROC curve for clinical event prediction were 0.80 for LV radial strain, 0.79 for LV circumferential strain, and 0.80 for LV longitudinal strain (Figure 3).

### Associations between independent variables in the initial CMR and clinical events

A multivariate analysis was performed to find the independent variables from among LV longitudinal strain, left ventricular ejection fraction and late gadolinium enhancement area. The only variable that maintained correlation with long-term combined events was LV longitudinal strain (HR = 1.282, CI 95%: 1.022–1.524, *p* = 0.007), while ejection fraction (HR = 0.924, CI 95%: 0.862–1.024, *p* = 0.723) and late gadolinium enhancement area (HR = 1.226, CI 95%: 0.932–1.378, *p* = 0.448) did not show a positive result.

## Subgroup analysis

### Study population

A total of 36 patients underwent a second CMR during the study follow-up and were included in the subgroup analysis. An increase in LVEF was observed in 20 patients. The baseline characteristics of these patients are presented in Supplementary Table S1. Overall, the mean age was 37.0 ± 17.2 years, and 77.8% were male. The most prevalent cardiovascular risk factor was systemic arterial hypertension, in 16.7% of patients. The mean sensitive cardiac troponin level was 22,400.0 ± 20,300.0 ng/L, and the median LVEF by CMR was 56.0 (14.7%).

### Association between myocardial strain at the initial CMR and LVEF improvement

In the univariate analysis, LV radial strain, LV circumferential strain, LV longitudinal strain, and RV longitudinal strain were positively associated with LVEF (Table 4). In the adjusted model, these associations lost statistical significance.

**Table 4:** Univariate and multivariate associations between myocardial strain measures and increased LVEF in 36 patients with acute myocarditis.

	Increase in the LVEF							
	Univariate analysis					Mutivariate analysis		
	Yes	No	HR	CI 95%	*p*	HR	CI 95%	*p*
LV radial strain (%)	19.4 ± 6.9	24.9 ± 7.9	0.896	0.804–0.999	0.032	0.975	0.909–1.046	0.487
LV circumferential strain (%)	−12.3 (3.9)	−15.8 (3.0)	1.406	1.060–1.865	0.014	1.022	0.901–1.158	0.738
LV longitudinal strain (%)	−11.4 ± 4.1	−14.7 ± 2.9	1.315	1.041–1.661	0.010	1.036	0.887–1.209	0.654
RV radial strain (%)	25.9 ± 12.6	30.4 ± 18.5	0.980	0.937–1.025	0.392			
RV circumferential strain (%)	−9.3 ± 3.6	−11.3 ± 3.0	1.213	0.976–1.508	0.073			
RV longitudinal strain (%)	−16.0 ± 4.3	−19.1 ± 4.1	1.197	1.005–1.426	0.035	1.020	0.900–1.156	0.760

Variables described as mean ± standard deviation or median (interquartile range); LVEF, left ventricle ejection fraction; LV, left ventricle; RV, right ventricle; HR, hazard ratio; CI, confidence interval.

### Myocardial strain at the initial CMR vs. at the follow-up CMR

**Figure 4:**
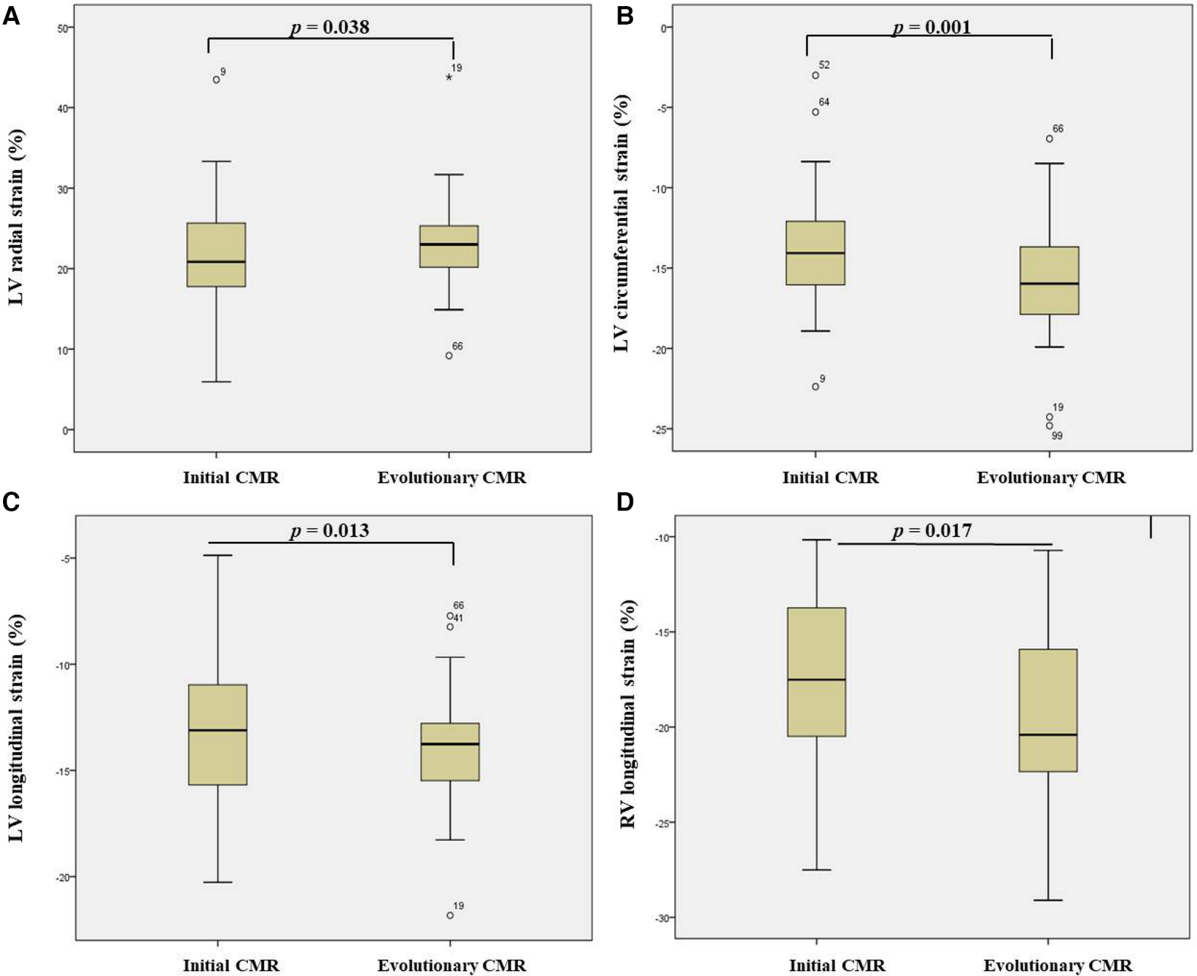
Boxplot diagrams presenting differences between myocardial strain in initial vs. evolutionary CMR: (**A**) LV radial strain; (**B**) LV circumferential strain; (**C**) LV longitudinal strain related and; (**D**) RV longitudinal strain. CMR, cardiac magnetic resonance; LV, left ventricle; RV, right ventricle.

LV radial strain, LV circumferential strain, LV longitudinal strain, and RV longitudinal strain increased significantly in the follow-up CMR compared with the initial CMR (Table 5 and Figure 4).

**Table 5:** Comparisons of myocardial strain measures between the initial CMR and the follow-up CMR.

	Initial CMR	Follow-up CMR	*p*
LV radial strain (%)	21.9 ± 7.8	23.0 ± 6.9	0.038
LV circumferential strain (%)	−14.1 (3.9)	−16.1 (3.9)	0.001
LV longitudinal strain (%)	−12.9 ± −3.9	−14.2 ± 3.2	0.013
RV radial strain (%)	27.9 ± 15.4	31.1 ± 13.6	0.232
RV circumferential strain (%)	−10.2 ± 3.5	−11.3 ± 4.9	0.063
RV longitudinal strain (%)	−17.4 ± 4.4	−19.4 ± 4.6	0.017

Variables described as mean ± standard deviation or median (interquartile range); CMR, cardiac magnetic resonance; LV, left ventricle; RV, right ventricle.

## Discussion

Our study has three main findings. First, measures of myocardial peak strain by CMR were significantly associated with the occurrence of clinical events in patients with AMC. Second, LV and RV strain were not associated with an increase in LVEF in the subgroup of patients who underwent a second CMR during follow-up. Furthermore, LV longitudinal strain was an independent variable correlated with prognosis in patients with AMC, even when analyzed together with ejection fraction and delayed enhancement. The only variable that maintained its correlation with the long-term composite event was LV longitudinal strain, while ejection fraction and late gadolinium enhancement area did not show a positive result. Finally, when the initial CMR findings were compared with the follow-up CMR findings, improvements in the measures of LV and RV myocardial strain were observed.

Several studies have investigated the prognostic value of CMR findings in patients with AMC (16–25). In a study by Fischer et al. (21), 455 patients with AMC were followed for 3.9 years. Clinical outcomes (hospitalization for HF, sustained ventricular tachycardia and death) occurred in 16% of patients. LV longitudinal strain was independently associated with adverse cardiovascular outcomes. Our results are in line with that study, as we showed that LV radial, circumferential and longitudinal strain were significant predictors of long-term clinical events. Our study simultaneously analyzed radial, circumferential and longitudinal strains of the LV and RV in AMC patients. However, strain in the RV was not associated with long-term prognosis.

Radial tissue strain was the only variable associated with an improvement in LVEF in a study of 37 AMC patients (18). Luetkens et al. (20) evaluated LV and RV tissue strain in 69 patients with AMC. The study showed that LVEF and RVEF significantly improved over the follow-up, as did both LV and RV strain (radial, circumferential and longitudinal), but only longitudinal LV strain was an independent predictor of LVEF improvement (20). In our study, radial, circumferential and longitudinal LV strain were associated with increased LVEF in the univariate analysis, but this association lost statistical significance in the multivariable analysis. This finding may be explained, at least in part, by the limited number of patients who underwent a follow-up CMR.

Specifically regarding RV, in 2,022 patients with AMC, a retrospective study showed that global circumferential, radial and longitudinal strain had a significant correlation with clinical events over a follow-up of 5.5 years. In contrast, all strain analyses in VR were not significant (24). Our results reinforce those findings, but in a prospective study.

A retrospective study showed that patients with AMC presented with lower longitudinal strain and circumferential strain than healthy individuals (16). Among those with preserved LVEF, no significant differences were seen in strain measures compared with healthy individuals. Similarly, Gatti et al. (17) compared 30 patients with AMC and preserved LVEF with healthy controls. The study failed to show any significant differences in LV strain measures between the two groups (17). In our subpopulation who underwent a follow-up CMR, the strain measures significantly improved over time, even among those with preserved LVEF at baseline.

In patients with preserved ejection fraction, a recent study evaluated 108 patients with suspected AMC who did not meet the definitive criteria and showed that longitudinal strain correlated with events only in patients with late gadolinium enhancement (22). Following the same line, including only patients with preserved ejection fraction, another retrospective study showed that global longitudinal strain provided independent prognostic value over late gadolinium enhancement characterization (23). A similar finding was obtained in our study, showing LV longitudinal strain as an independent variable correlated with the patient's prognosis.

Our results should be interpreted in light of some limitations. This study was a prospective observational single-center study of patients with AMC. A control group was not included, and the results may not be generalizable to other populations. Ventricular arrhythmias were not included as an outcome because they were not observed in this series. Hyperemia and mapping sequences were not evaluated (because of the timeframe of the patients). CMR-based diagnosis of myocarditis has evolved since the original enrollment of patients. The LGE area was calculated with 2 SD, which in a way may overestimate the impairment. In patients in whom edema was not visualized in the CMR, no diagnostic auxiliary imaging method or endomyocardial biopsy was performed. Finally, different treatments were administered to patients with AMC during the study period, which could have influenced the results. Further studies with larger sample sizes may further validate our findings.

In conclusion, measures of myocardial strain obtained by CMR can provide prognostic information in patients with AMC. LV radial, circumferential and longitudinal strain were associated with long-term clinical events in these patients. Although LGE had not shown a predicted value for event over the strain in our manuscript, additional studies are needed to better understand LGE predicted value in this scenario.

## Data Availability

The raw data supporting the conclusions of this article will be made available by the authors, without undue reservation.
