# Supplementary material for: The association of myocardial strain with cardiac magnetic resonance and clinical outcomes in patients with acute myocarditis
Source: Front Cardiovasc Med. 2023 Jul 31;10:1121083. doi: 10.3389/fcvm.2023.1121083 (PMC10425551; doi:10.3389/fcvm.2023.1121083)
Supplement: Supplementary file 1 [file Table1.docx]

|  |  | Increase in the LVEF | |  |
| --- | --- | --- | --- | --- |
| Baseline characteristics | Overall (n=36) | Yes (n=20) | No (n=16) | *p* |
| Age | 37.0 + 17.2 | 35.1 + 13.9 | 39.4 + 20.8 | 0.46 |
| Male sex (%) | 28 (77.8%) | 15 (75%) | 13 (81.3%) | 0.654 |
| Diabetes Mellitus (%) | 0 | 0 | 0 | NS |
| Hypertension (%) | 6 (16.7%) | 4 (20%) | 2 (12.5%) | 0.549 |
| Smoking (%) | 5 (13.9%) | 3 (15%) | 2 (12.5%) | 0.829 |
| Dyslipidemia (%) | 5 (13.9%) | 3 (15%) | 2 (12.5%) | 0.829 |
| Troponin (ng/L) | 22400 + 20300 | 28600 + 22100 | 14600 + 15000 | 0.038 |
| Creatinine(mg/dL) | 1.0 + 0.2 | 1.1 + 0.2 | 0.9 + 0.2 | 0.376 |
| LVEF (%) | 56.0 (14.7) | 44.0 (14.9) | 59.8 (8.6) | <0.0001 |
| CRP (mg/dL) | 49.5 + 62.8 | 50.3 + 50.9 | 48.6 + 76.8 | 0.937 |
| BNP (pg/mL) | 438.3 + 716.7 | 803.5 (903.7) | 75.2 (88.3) | 0.094 |
| **Medications used at hospital discharge** | | |  |  |
| Beta-blocker (%) | 20 (55.6%) | 12 (60%) | 8 (50%) | 0.549 |
| Spironolactone (%) | 11 (30.6%) | 9 (45%) | 2 (12.5%) | 0.035 |
| NSAID (%) | 12 (33.3%) | 6 (30%) | 6 (37.5%) | 0.625 |
| Colchicine (%) | 9 (25%) | 4 (20%) | 5 (31.3%) | 0.439 |
| ACEI /ARB (%) | 24 (66.7%) | 17 (85%) | 7 (43.8%) | 0.009 |

**Supplemental Table1.** Clinical characteristics of patients according to the presence of an increase in LVEF in the subgroup who underwent a follow-up CMR

**Legend:** Variables described as mean + standard deviation or median (interquartile range); LVEF=left ventricle ejection fraction by CMR; CRP=C-reactive protein; BNP=B-type natriuretic peptide; NSAID=nonsteroidal anti-inflammatory; ACEI=angiotensin converting enzyme inhibitors; ARB=angiotensin receptor blockers; NS=non significant.
